# Immunity Against *Mycobacterium avium* Induced by DAR-901 and BCG

**DOI:** 10.3390/vaccines13060619

**Published:** 2025-06-07

**Authors:** Getahun Abate, Krystal A. Meza, Chase G. Colbert, Octavio Ramos-Espinosa, Nancy J. Phillips, Christopher S. Eickhoff

**Affiliations:** 1 Division of Infectious Diseases, Allergy and Immunology, Department of Internal Medicine, Saint Louis University, St. Louis, MO 63104, USAoctavio.ramos@health.slu.edu (O.R.-E.);; 2 Department of Pathology, Saint Louis University, St. Louis, MO 63104, USA

**Keywords:** *M. avium*, BCG, DAR-901, immunity, BALB/c

## Abstract

**Background:** The prevalence of pulmonary nontuberculous mycobacteria (NTM) is increasing in Europe and North America. Most pulmonary NTM cases are caused by *Mycobacterium avium* complex (MAC). The treatment of pulmonary MAC is suboptimal with failure rates ranging from 30% to 40% and there is a need to develop new vaccines. **Methods**: We tested the ability of two whole-cell vaccines, DAR-901 (heat-killed *M. obuense*) and BCG (live-attenuated *M. bovis*), to induce MAC cross-reactive immunity by first immunizing BALB/c mice and then performing IFN-γ ELISPOT assays after overnight stimulation of splenocytes with live MAC. To study the ability of these vaccines to protect against MAC infection, BALB/c mice were vaccinated with DAR-901 (intradermal) or BCG (subcutaneous or intranasal) and challenged with aerosolized MAC 4 weeks later. A group of mice vaccinated with BCG were also treated with clarithromycin via gavage. Lung colony-forming units (CFU) in immunized mice and unvaccinated controls were quantified 4 weeks after infection. Histopathology was used to quantify lung inflammation and flow cytometry was used to study lung immunity in BCG-vaccinated and unvaccinated mice following MAC infection. To increase the safety profile of mucosal BCG vaccination, we studied BCG with a “kill switch” (tetR BCG) in *scnn1b*-transgenic mice (i.e., mice prone to cystic fibrosis-type lung diseases). **Results**: Our results showed that (i) DAR-901 induced cross-reactive immunity to MAC to a similar level as BCG, (ii) DAR-901 and BCG protected against aerosol MAC challenge, (iii) mucosal BCG vaccination, compared to systemic BCG and DAR-901 vaccinations, provided the best protection against MAC challenge, (iv) BCG vaccination did not interfere with anti-MAC activities of clarithromycin, (v) BCG-vaccinated mice had increased inflammation and increased frequencies of activated CD4 and CD8 T cells following MAC infection, and (vi) doxycycline treatment of tetR BCG-vaccinated mice decreased lung BCG CFU without affecting MAC immunity. **Conclusions**: Both DAR-901 and BCG vaccinations induce MAC cross-reactive immunity and protect against aerosolized MAC challenges. Mucosal BCG vaccination provides the best protection and TetR BCG could enhance the safety of mucosal BCG vaccination.

## 1. Introduction

Nontuberculous mycobacteria (NTM) are all mycobacteria except *Mycobacterium tuberculosis* (Mtb) complex and *M. leprae*. There are more than 190 NTM species and subspecies but only a few are clinically relevant [1]. NTM can affect any organ in the body, but the pulmonary form is the most common form in patients who are negative for human immunodeficiency virus (HIV) [2]. In the US and Western Europe, the prevalence of pulmonary NTM in HIV-negative patients has been increasing over the last decade [2,3,4,5,6,7,8]. The reasons for these recent increases in the prevalence of pulmonary NTM are not clearly known but advances in extending the life expectancy of patients with underlying chronic lung diseases such as cystic fibrosis (CF) and COPD, and wide application of immunosuppressive medications in medicine may have played a role [9,10,11,12,13].

In North America and parts of Europe, *M. avium* complex (MAC) is the most common NTM species associated with pulmonary NTM [8,14]. Regardless of the causative NTM species, management of pulmonary NTM is extremely difficult. Treatment requires the use of multiple drugs for at least 12 months from the first culture-negative results which in most cases means more than 18 months of treatment [15]. Despite treatment for several months, failure rates in the range of 30–40% have been reported [14,16,17]. Thus, there is an urgent need to develop new vaccines and new therapeutics. This urgency was emphasized in the last NTM workshop organized by the National Institute of Allergy and Infectious Diseases [18].

In the last decade, significant advances have been made in understanding tuberculosis (TB) immunology and bringing new vaccines as well as immunotherapeutics to clinical trials. Lessons learned in the TB field could be very useful for work on pulmonary MAC. Like Mtb, MAC is an intracellular pathogen, and its control relies primarily on mounting effective cell-mediated immunity [19,20]. Subunit vaccines and RNA vaccines have been tested in mice by other groups [21,22,23,24,25]. Mycobacterial whole-cell vaccines induce robust immune responses and they are among promising vaccine candidates for mycobacterial diseases [26,27]. This study was performed with the objective of testing two whole-cell vaccines, DAR-901 (heat-killed whole-cell *M. obuense*) and bacillus Calmette Guerin (BCG) to induce MAC-specific immunity and evaluate their potential for prevention of pulmonary MAC in a murine model. We and others previously showed that BCG induces MAC cross-protective immunity in mice and humans [21,23,28]. However, advancing a live-attenuated vaccine for human use in patients with underlying lung diseases who are at high risk of developing pulmonary MAC has become challenging because of safety concerns. Therefore, in this study, we also included a tetR BCG with a “kill switch” which allows for replication of the mycobacteria in vivo for a controlled period of time.

## 2. Materials and Methods

Reagents and Bacterial culture. MAC (*M. avium*, ATCC 700898) and Tice bacillus Calmette-Guerin (BCG, Merck, Rahway, NC, USA) were used. Large bacterial lots of MAC and BCG were grown in roller bottles at 37 °C in albumin-dextrose-catalase (ADC)-supplemented Middlebrook 7H9 broth, aliquoted in 1 mL volume and stored at −80 °C. TetR BCG containing two plasmids with lysin genes from L5 and D29 mycobacteriophages obtained from Dr. Schnappinger’s group (Cornell University) [29] was used in our experiments. These plasmids contain lysin genes from L5 and D29 mycobacteriophages cloned downstream of a Tet repressor (TetR) tsc10-regulated, tetracycline-inducible promoter Pmyc1tetO. Addition of tetracyclines alleviates the repression by TSC10 and allows for lysin induction which leads to lysis of the mycobacteria.

DAR-901, a vaccine comprised of heat-inactivated *Mycobacterium obuense*, was used as one of the vaccines. DAR-901 in 2 mL vials containing 0.3–0.4 mL of a 1 mg/mL suspension of heat-inactivated organisms was kindly provided by Dr. Fordham von Reyn (Geisel School of Medicine at Dartmouth, Hanover, New Hampshire). It was previously reported that a 1 mg DAR-901 contains 7 × 10^6^ colony-forming units (CFU) and 0.3 mg contains 2 × 10^6^ CFU bacteria [30,31].

Animals. Animal experiments were reviewed and approved by the Saint Louis University Institutional Animal Care and Use Committee (IACUC). Animal experiments were performed with 6- to 8-week-old BALB/c mice as described previously [32]. *Scnn1b*-transgenic mice on the C57BL/6 background were obtained from Dr. Matthew Wolfgang (University of North Carolina). Only mice confirmed to be transgenic by genome sequencing were used. *Scnn1b*-transgenic mice overexpress the beta subunit of ENaC (gene *Scnn1b*) under the control of the club cell secretory protein promoter. These mice show increased sodium absorption in the airways, leading to airway surface liquid dehydration, mucus obstruction, and neutrophilic inflammation similar to pathological findings in CF patients [33,34]. All mice were euthanized by placing them in a designated chamber and introducing CO_2_ from a compressed gas source at a displacement rate of 30–70% chamber volume per minute.

Vaccination and routes of administration. Supplementary Table S1 summarizes vaccinations including routes and doses; other interventions and MAC challenges used in different experiments. BCG was administered intranasally (IN) to target the lungs and mucosal immunity, subcutaneously (SC) or intradermally (ID). Aliquots of BCG were thawed and pelleted by centrifuging at 1684× *g* force for 15 min at 4 °C. Pellets were resuspended in phosphate buffer saline (PBS). Mice that received IN BCG had 1 × 10^7^ BCG delivered in 40 µL doses split between nostrils. Groups of mice that received SC BCG received 1 × 10^7^ bacteria in 100 µL to base of tail. Mice that received ID BCG had 1 × 10^7^ in 50 µL PBS delivered to the left side of shaved abdomen. The 1 × 10^7^ IN dose was used based on data from a previous publication [35], and we used a similar dose for systemic vaccination to avoid differences in immunity based on administered dose. DAR-901 was diluted to various doses (0.3, 1, and 2 mg/dose) in PBS and administered in a total volume of 50 µL using insulin syringes (BD Biosciences, San Jose, CA, USA). TetR BCG suspended in PBS was used at the same dose IN and half the mice which received TetR BCG were treated with doxycycline (1 mg/mL in the drinking water) for 2 weeks starting 2 weeks after vaccination. DAR-901 suspensions were prepared in PBS and ID doses were administered at alternating sites on the stomach of anesthetized mice every one to two weeks up to a total of three doses. A one-week interval between doses of DAR-901 was used in the initial experiments studying DAR-901’s ability to induce MAC cross-reactive immunity. The interval was changed to two weeks for MAC challenge studies because a two-week interval dosing was shown to be protective in TB-infected mice previously [36]. We tested mucosal and systemic BCG vaccination. Vaccinated mice received only one type of vaccination. The ID route was used as a systemic route of BCG vaccination only in experiments when ID DAR-901 was used. Mice were anesthetized with ketamine/xylazine cocktail (60 mg/kg and 5 mg/kg, respectively) intraperitoneally before IN or ID vaccination. In experiments performed to measure vaccine-induced immunity, BCG or DAR-901 immunized mice were euthanized 4 weeks after the final vaccination (outlined in Supplementary Figure S1) and splenocytes were used for IFN-γ ELISPOT assays as described below.

Measuring vaccine-induced MAC immunity. We used IFN-γ ELISPOT assays to measure MAC-reactive T cell immunity. The assay was conducted as described previously [28]. Briefly, mice were euthanized at different time points and splenocytes (5 × 10^5^ cells/well) were stimulated overnight with live BCG at a multiplicity of infection (MOI) of 3, *M. avium* at MOI of 3, or DAR-901 at 2 and 10 µg/mL. Cells resting in media alone were used as negative controls. After overnight incubation at 37 °C, ELISPOT plates were developed using biotinylated anti-IFN-γ (BD Biosciences clone XMG1.2), streptavidin horseradish peroxidase (SA-HRP; Jackson ImmunoResearch, West Grove, PA, USA), and AEC development solution per manufacturer recommendations (BD Biosciences). IFN-γ producing spots in each well were enumerated using a C.T.L. (ImmunoSpot analyzer, Shaker Heights, OH, USA) and software (ImmunoCapture Version 6.6, Bio-Techne Corporation, Minneapolis, MN, USA). The results are presented as spot-forming cells (SFC, mean ± SE) per million splenocytes.

MAC challenge. In experiments performed to study the protective effects of vaccinations (i.e., BCG, TetR BCG, or DAR-901), mice were challenged with aerosolized MAC 4 weeks after vaccination (outlined in Supplementary Figures S2 and S3). Briefly, three weeks before aerosol challenges, aliquots of MAC (ATCC 700898) were thawed and cultured in fresh ADC (Sigma, St. Louis, MO, USA)-supplemented 7H9 media (Fisher Scientific, St. Louis, MO, USA) without Tween. On the day of the challenge, mycobacterial suspensions were centrifuged, and pellets were resuspended in PBS. The optical density (OD) of the suspension was measured at 600 nm. MAC isolated from the lungs of infected mice (i.e., passaged once) were used for challenge. Bacterial suspension was diluted to adjust the OD to 0.7. We estimated 3.1 × 10^7^ CFU per OD unit based on results from previous titration experiments. MAC at an estimated final concentration of 2 × 10^7^ CFU/mL was added to the nebulizer and delivered via the aerosol route using Glas-Col Inhalation Exposure System (IES) (Glas-Col Inc., Terre Haute, IN, USA) using settings described previously [37]. Animals euthanized immediately post-exposure were used to quantify the delivery dose using methods described above.

Quantifying lung CFU after MAC infection. Mice were infected with aerosolized MAC. A total of 3–7 mice were sacrificed on day 0 and in weeks 2, 4, 6, and 8 after infection. Lungs were homogenized in sterile PBS using a bead mill homogenizer, serially diluted, and plated in duplicate for CFU quantification on 7H11 agar media. For mice vaccinated with BCG, additional oleic-albumin-dextrose-catalase (OADC)-supplemented Middlebrook 7H10 agar media containing isoniazid at a concentration of 1 µg/mL was used to inhibit the growth of BCG. Plates cultured at 37 °C were read every week and the CFU counts were finalized on week 4.

To study the effects of BCG vaccination on anti-MAC activities of clarithromycin, BCG-vaccinated and unvaccinated mice were infected with aerosolized MAC. Two weeks after infection, 11 mice were treated with clarithromycin at a concentration of 100 mg/kg for four weeks before they were euthanized (Supplementary Table S1). Clarithromycin was administered in a 0.2 mL volume by esophageal cannula (gavage) 5 days per week. Percent inhibition was calculated as follows: % inhibition = 100 − [100 × (CFU in mice with intervention/mean CFU in mice without intervention)]. Intervention includes BCG vaccination and/or clarithromycin treatment.

Measuring recruitment of T cells to the lungs following BCG vaccination. At different time points post-vaccination, lungs were extracted, homogenized, and digested with collagenase/DNase. Then, single-cell suspensions were prepared for flow cytometric studies. At least 10,000 events were acquired, lymphocytes gated based on side scatter and forward scatter, and live CD3^+^ T cells gated. Frequencies of specific T cell subsets were analyzed. Percent CD4 and CD8 T cells that were CD44^+^ and/or CXCR3^+^ were quantified.

Lung histopathology. The lungs of mice were perfused with 10% formalin dissolved in phosphate buffer saline (PBS) via the trachea, the left lung immersed in the same fixative and embedded in paraffin. Five-micrometer sections were stained with hematoxylin and eosin (H and E). Slides were scanned using the VS110 Slide Scanning System (Olympus America, Center Valley, PA, USA) and digitized with the VS-ASE (Olympus software version 2.9). Images were analyzed using QuPath-Open Software for Bioimage Analysis [38]. Percent inflamed area was calculated as follows: Percent inflammation area = [Total areas of inflammation (µm^2^) ÷ total lung area (µm^2^)] × 100. Additional images were obtained directly from a microscope and camera (Olympus BX53, Olympus UC90, Center Valley, PA, USA).

*Statistical analysis.* Mean, medians, and percentages were used to describe assay results. IFN-γ ELISPOT results and lung CFU were described as mean or median numbers ± standard error (SE) of IFN-γ SFC. The results between groups were compared using a nonparametric *t*-test. Percent lung CD4 and CD8 T cells expressing CD44 and/or CXCR3 obtained by flow cytometry and percent inflammation areas on histology following MAC infection in BCG-vaccinated and unvaccinated mice were compared using a nonparametric *t*-test.

## 3. Results

**Figure 1:**
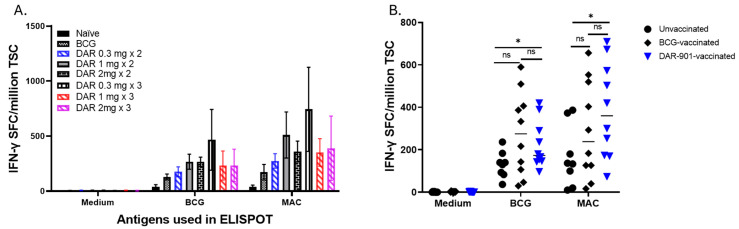
*M. avium* cross-reactive immunity induced by vaccination with BCG and DAR-901. (**A**) shows the number of IFN-γ spot-forming cells (SFC) in mice (4 mice per group) vaccinated with BCG or 2 and 3 doses of 0.3 mg, 1 mg, or 2 mg DAR-901 a week apart. Six- to eight-week-old female BALB/c mice were vaccinated with BCG (1 × 10^7^ intradermal) or different concentrations (0.3 mg, 1 mg, and 2 mg) of DAR-901 (2 or 3 intradermal doses a week apart). Four weeks after last vaccination, mice were euthanized and splenocytes were used in IFN-γ ELISPOT assays. Here, splenocytes (5 × 10^5^ cells/well) were stimulated overnight with live BCG at a multiplicity of infection (MOI) of 3, *M. avium* at an MOI of 3, or media alone as a negative control. IFN-γ producing spots in each well were enumerated using a C.T.L. ImmunoSpot analyzer and software and results are presented as SFC (mean ± SE) per million total splenic cells (TSC). Both BCG and different doses of DAR-901 induced BCG-reactive and MAC cross-reactive immunity. (**B**) Shown are results from experiments using larger numbers of BALB/c mice (10 mice per group) vaccinated with BCG (ID), 2 doses of 0.3 mg DAR-901 a week apart or left unvaccinated. DAR-901 (2 doses of 0.3 mg) induced BCG-reactive and MAC-reactive immunity similar to immunity induced by BCG. The lines show median values for each group. MAC cross-reactive immunity induced by DAR-901 was significantly higher than the unvaccinated group (*, *p* = 0.03 for BCG stimulation and *p* = 0.034 for MAC stimulation, Mann–Whitney U test). ns, not significant.

DAR-901 and BCG induce MAC cross-reactive immunity. DAR-901, like BCG, is a whole-cell mycobacterium. Unlike BCG, DAR-901 contains a killed NTM. We used the intradermal route for DAR-901 administration because that was the route used in animal and human studies for TB immunity or protection [31,36]. Similarly, we tested 2 and 3 doses of DAR-901 vaccination since multiple doses were required in previous works to enhance TB immunity [30,31,36]. In our initial experiments, we compared 2 and 3 injections of three different concentrations of DAR-901 ranging from 0.3 mg to 2 mg (Supplementary Figure S4). Figure 1A shows that both 2 and 3 doses of DAR-901 induce robust BCG and MAC-cross-reactive T cell immunity. Immunity to BCG induced by the different doses of DAR-901 was similar to the MAC immunity they induced. The highest level of IFN-γ SFC was obtained with three doses of 0.3 mg DAR-901 with 466 ± 276 IFN-γ SFC (mean ± SE) after in vitro BCG stimulation and 743 ± 382 IFN-γ SFC (mean ± SE) after in vitro MAC stimulation of splenocytes. Subsequently, we compared MAC cross-reactive immunity induced by DAR-901 with immunity induced by BCG administered via the same route. Figure 1B shows that DAR-901 and BCG vaccines induced similar levels of MAC immunity as BCG. However, only DAR induced significantly higher MAC-specific immunity compared to unvaccinated mice (*p* = 0.034). The two vaccines induce similar levels of MAC immunity 291.7 ± 72.3 IFN-γ SFC/million spleen cells (mean ± SE) in the BCG-vaccinated group vs. 384.6 ± 70.8 (mean ± SE) for the DAR-901 vaccinated group (*p* = 0.31, Mann–Whitney U test). The two vaccines also induced similar levels of BCG-immunity with 276.7 ± 62.2 IFN-γ SFC/million spleen cells (mean ± SE) for BCG vs. 222 ± 34.8 (mean ± SE) for DAR-901 (*p* = 0.81, Mann–Whitney U test).

**Figure 2:**
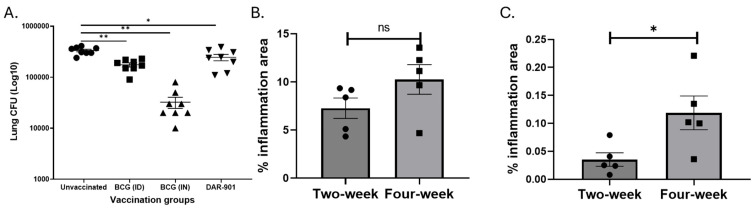
Effects of vaccinations on the growth of *M. avium* and inflammation in lungs. (**A**) Female BALB/c mice (6–8 weeks old, *n* = 8 per group) were vaccinated with BCG ID (1 × 10^7^ bacteria in 100 µL to base of tail), BCG IN (1 × 10^7^ CFU total in both nostrils) or DAR-901 (2 doses of 0.3 mg ID, 2-weeks apart). Four weeks after last vaccination, mice were infected with aerosolized MAC delivered from a dose jar with a concentration of 2 × 10^7^ CFU/mL. Mice were euthanized 4 weeks after infection and MAC lung CFU were quantified by culturing on 7H10 media. The figure shows medians (lines). Lung CFU was significantly lower in mice vaccinated with ID BCG (*p* < 0.001, *t*-test), IN BCG (*p* < 0.001), and two doses of 0.3 mg DAR-901 (*p* <0.01). The results for BCG SC were similar to BCG ID. (**B**,**C**) show the percent lung inflammation in BCG-vaccinated and unvaccinated mice, respectively. BALB/c mice (5 per group per timepoint) were vaccinated with BCG IN (1 × 10^7^ CFU total) or kept unvaccinated. Half the mice were female. Four weeks after vaccination, all mice were infected with aerosolized MAC as above. At 2 and 4 weeks after infection, the left lung was harvested from each mouse and kept in formalin. Paraffin-embedded sections were stained with hematoxylin and eosin stain. Lung areas affected by any type of inflammation were divided by the total lung area to obtain percent inflammation. (**B**) Inflammation in BCG-vaccinated mice 2- and 4-weeks after MAC infection. At 2 and 4 weeks post-MAC infection, percent inflammation in vaccinated mice were 7.3 ± 1.1 and 10.3 ± 1.5 (mean ± SE), respectively. At both time points, BCG-vaccinated mice had significantly higher percent inflammation compared to unvaccinated mice (*p* = 0.0079). The percent inflammation did not significantly change between 2 and 4 weeks after infection with *M. avium* (*p* = 0.09). (**C**) Inflammation in unvaccinated mice. Percent inflammation in unvaccinated mice significantly increased from 0.035 ± 0.012 at 2 weeks to 0.12 ± 0.03 (mean ± SE) at 4 weeks after MAC infection (*p* = 0.03, Mann–Whitney U test). *, *p* < 0.05; **, *p* < 0.01. ns, not significant.

DAR-901 and BCG protect against MAC aerosol challenges in mice. We optimized a murine model of MAC infection to study the relevance of vaccine-induced immunity for MAC protection. After an aerosol infection, MAC grows and reaches its peak after 4 weeks (Supplementary Figure S5). We used mouse-adapted MAC (i.e., MAC passaged in mice lung at least once). Figure 2A shows DAR-901, systemic BCG, and mucosal BCG vaccinations protect against aerosol MAC challenge (*p* < 0.001). Mucosal BCG vaccination led to a 1.5 log decrease in lung MAC CFU whereas systemic BCG vaccination and DAR-901 vaccination provided a 0.5 and 0.6 log reduction in CFU, respectively. These results indicate that mucosal BCG vaccination provides the best protection compared to systemic BCG and DAR-901 vaccinations.

**Figure 3:**
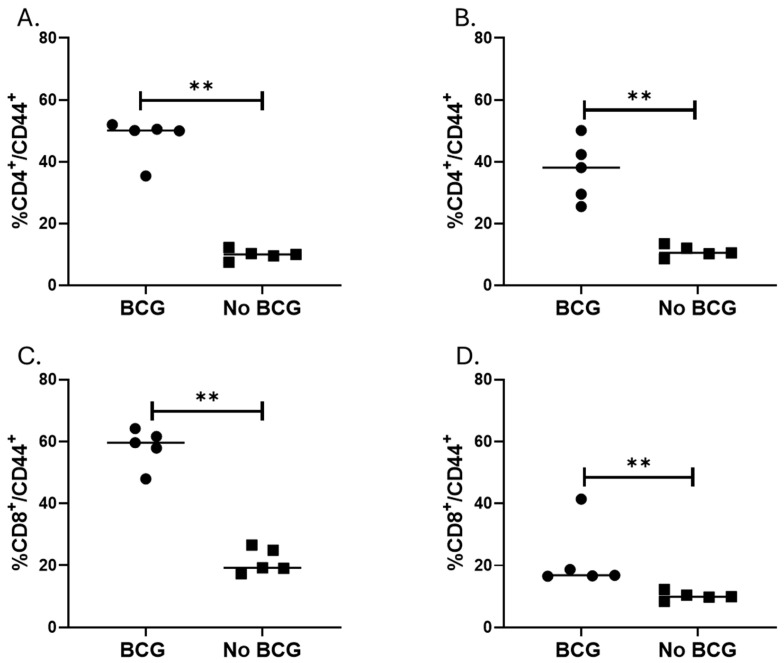
Effector CD4 and CD8 T cells in the lung after aerosol MAC infection of vaccinated and unvaccinated mice. BALB/c mice vaccinated (or not) with IN BCG were infected with aerosolized MAC 4 weeks after vaccination. Two- and four weeks post-infection (i.e., days 42 and 56 post-vaccination), lung tissue was homogenized, and cells were stained with live/dead, CD3, CD4, CD8, CD44, and CXCR3 antibodies. More than 10,000 events were acquired on flow cytometry. (**A**,**B**) show percent CD4/CD44 T cells in vaccinated and unvaccinated mice on days 42 and 56, respectively. (**C**,**D**) show percent CD8/CD44 T cells in vaccinated and unvaccinated mice on days 42 and 56, respectively. The results show that vaccinated mice had significantly higher percent CD4^+^/CD44^+^ and CD8^+^/CD44^+^ on days 42 and 56 with *p* = 0.0079 for both time points. **, *p* < 0.01.

Mucosal BCG vaccination increases inflammation with an increased percentage of activated T cells. Because mucosal BCG vaccination provided the best protection from MAC, we assessed histopathological changes after MAC infection of unvaccinated and BCG (IN) vaccinated BALB/c mice. On weeks 2 and 4 post-MAC infection, BCG-vaccinated mice had significantly higher levels of inflammation compared to unvaccinated mice (*p* = 0.0079, Mann–Whitney U test). Total areas of inflammation significantly increased in unvaccinated but not BCG-vaccinated mice at 4 weeks post-infection compared to inflammation at week 2 (*p* = 0.03, Figure 2B,C). To identify primary cells which may be involved in the inflammation, we performed flow cytometric analysis of cells from the lungs of BCG-vaccinated and unvaccinated mice 2 and 4 weeks after MAC infection. Percentages of CD4^+^ and CD8^+^ T cells expressing CD44 were higher in vaccinated mice 2 weeks and 4 weeks post-MAC infection (Figure 3, *p* = 0.0079). To further study whether these activated T cells are recruited or are activated in the bronchial mucosa, we analyzed cells for CXCR3. The results showed that BCG-vaccinated mice had significantly higher frequencies of CD4^+^CXCR3^+^ T cells compared to unvaccinated mice. BCG-vaccinated and unvaccinated mice did not differ in the percent of CD8^+^ T cells that are CD44^+^CXCR3^+^ (Supplementary Figure S6).

BCG-induced MAC immunity does not interfere with the antimycobacterial activities of macrolide. Macrolides have anti-inflammatory activities. Studies conducted on azithromycin, a preferred macrolide for MAC treatment, have shown that it inhibits the production of IFN-γ and IL-17, suppresses CD4 T cell activation, and inhibits the expansion of effector T cells [39,40,41]. It is not known if these immunosuppressive activities of macrolides interfere with potent MAC immunity induced by BCG. We tested the interaction between macrolide and BCG in BALB/c mice. The anti-MAC activities of clarithromycin in BCG-vaccinated BALB/c mice were similar to the activities in unvaccinated mice, indicating that BCG-induced MAC immunity did not interfere with the anti-MAC activities of clarithromycin (Supplementary Figure S7).

**Figure 4:**
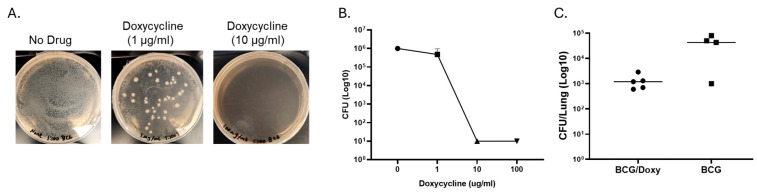
Effects of doxycycline on the growth of TetR BCG on 7H10 media, inside macrophages, and in mice. (**A**) shows effect of doxycycline on the growth of TetR BCG on 7H10 media containing different concentrations of doxycycline ranging from 0 to 10 μg/mL. (**B**) Effects of doxycycline on the intracellular growth of TetR BCG. Macrophages from three healthy volunteers were plated in 96-well plates at a concentration of ~10^4^ per well in triplicate and infected with TetR BCG. Doxycycline at concentrations ranging from 0 to 100 were added. After 72 h, macrophages were lysed, and residual BCG was quantified by culturing on 7H10 media. (**C**) Effects of doxycycline on the growth of TetR BCG in the lungs of mice. *Scnn1b*-transgenic mice were vaccinated with TetR BCG (IN, 1 × 10^7^ CFU). Two weeks after vaccination, five mice were treated with doxycycline (1 mg/mL in the drinking water) for two weeks, and other mice (*n* = 5) received no doxycycline. Four weeks after vaccination, numbers of BCG in the lung were quantified by culturing lung homogenate on 7H10 media. Doxycycline inhibits the in vitro and in vivo growth of TetR BCG.

TetR BCG provides the potential to limit the overgrowth of BCG without affecting MAC immunity. Vaccines targeting high-risk populations with structural lung diseases have to be safe. BCG is a live-attenuated mycobacterium and retains the ability to replicate. One approach to increase the safety of BCG is the use of BCG that has a “kill switch”. We used a tetR BCG that has two plasmids with lysin genes from L5 and D29 mycobacteriophages cloned downstream of a Tet repressor (TetR) tsc10-regulated, tetracycline-inducible promoter Pmyc1tetO. The addition of tetracyclines alleviates the repression by TSC10 and allows for lysin induction which leads to lysis of the mycobacteria. Figure 4 shows that treatment with doxycycline limits the growth of TetR BCG on 7H10 media (A), in vitro growth within macrophages (B), and in mice (C). When measured two weeks after a two-week treatment of mice with doxycycline following IN vaccination, lung CFU decreased by more than one log compared to the non-treated group.

**Figure 5:**
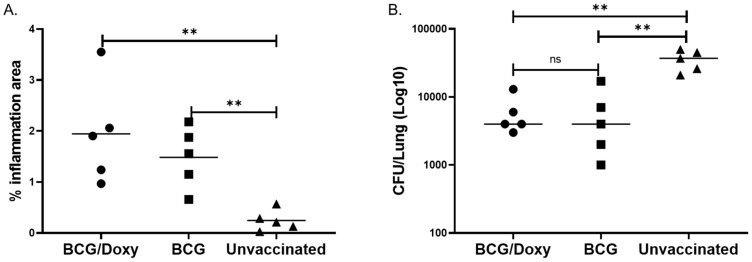
Effect of TetR BCG to protect *scnn1b*-transgenic mice from MAC infection. (**A**) Area of lung inflammation following aerosol MAC infection of vaccinated and unvaccinated mice. A total of 15 mice were used, 10 were vaccinated with tetR BCG via the IN route and 5 were unvaccinated. Five of the vaccinated mice received doxycycline through drinking water for two weeks starting two weeks after vaccination. All mice were challenged with aerosolized MAC (ATCC 700898, 2 × 10^7^) 4 weeks after vaccination. Four weeks after the MAC infection, the left lung was harvested, stained with H&E, and histopathological changes analyzed. The percentage area of inflammation was obtained by dividing the area of lung affected by inflammation by total lung area. Levels of inflammation were significantly higher in vaccinated mice (*p* = 0.0079, Mann–Whitney U test). Doxycycline-treated mice had significantly better inflammation compared to BCG-vaccinated mice but doxycycline untreated mice (*p* = 0.05, Mann–Whitney U test), indicating doxycycline treatment did not affect and even improve the ability of tetR BCG to induce inflammation. (**B**) MAC CFU in lungs of vaccinated and unvaccinated mice. Four weeks after vaccination, BCG numbers in the lungs were quantified by culturing lung homogenates on 7H10 media containing 1 µg/mL isoniazid or no isoniazid. Isoniazid-containing media was used to inhibit the growth of residual BCG. TetR BCG-vaccinated/doxycycline-treated, tetR BCG-vaccinated/not receiving doxycycline treatment, and unvaccinated groups had lung CFU (mean ± SE) of 6000 ± 1817; 6200 ± 2888, and 35,800 ± 5490, respectively, in isoniazid-containing media. The three groups of mice had a CFU (mean ± SE) of 12,200 ± 3720; 8200 ± 3121, and 43,800 ± 6320, respectively, in isoniazid-free media. TetR BCG-vaccinated mice, regardless of doxycycline treatment, had significantly lower lung CFU compared to unvaccinated mice in both isoniazid-free and isoniazid-containing media (*p* < 0.01). **, *p* < 0.01. ns, not significant.

**Figure 6:**
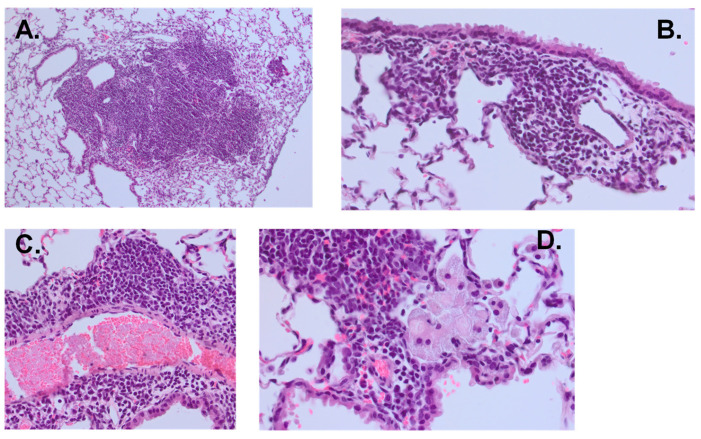
Lung histopathology from mice showing robust inflammation following MAC infection. All slides were stained with hematoxylin and eosin. (**A**) shows bulky peribronchial lymphocyte-predominant infiltrate (10×). (**B**) shows peribronchial lymphocyte-predominant infiltrate (40×). (**C**) shows perivascular lymphocyte-predominant infiltrate (40×). (**D**) shows foamy macrophages within an alveolar space adjacent to a peribronchial lymphocyte-predominant infiltrate (60×).

We next studied the effects of MAC immunity induced by tetR BCG in *scnn1b*-transgenic mice since they may be more vulnerable to MAC infection or progression because of CF-like changes in airways. *Scnn1b*-transgenic mice were vaccinated with tetR BCG (via IN route) or left unvaccinated. Half of the vaccinated mice received doxycycline treatment for two weeks starting two weeks after vaccination. At 4 weeks post-vaccination, all mice were challenged with aerosolized MAC. Four weeks after the MAC infection, lung histology was studied and lung CFU was quantified. In *scnn1b*-transgenic mice, lung inflammation is significantly higher in mice vaccinated with TetR BCG (Figure 5A) and tetR BCG-vaccinated mice had significantly lower lung MAC CFU post-infection (Figure 5B). Percent inflammation 4 weeks after MAC infection in unvaccinated *scnn1b* mice (Figure 5A) was not different from percent inflammation in wild-type BALB/c mice (Figure 2C) (*p* = 0.42). Doxycycline treatment did not affect the ability to cause inflammation in the lungs or provide protective immunity. Histopathological results described as inflammation include peribronchial inflammation, perivascular inflammation, and the presence of foamy macrophages with lymphocytes (Figure 6).

## 4. Discussion

BCG and DAR-901 are able to induce MAC cross-reactive immunity. We and other groups have shown that BCG induces MAC cross-reactive immunity in animals and humans [21,23,28]. This is the first study that compared MAC-specific immunity induced by BCG with immunity induced by DAR-901. Our results, using the same systemic route of administration, clearly showed that DAR-901 induced the same level of MAC T cell immunity as BCG. In addition, DAR-901 provided the same protection against MAC as systemic BCG vaccination, but the level of protection was significantly lower compared to mucosal BCG vaccination. In our study, we used a very high challenge dose of MAC for mice infection. The environment (e.g., water, soil) is the likely source of MAC infections, and the concentrations of MAC in the environment are expected to be much lower than what was used in our experiments [42,43,44]. Therefore, our results showing the ability of DAR-901 to inhibit MAC after challenge with a high concentration of aerosolized MAC are very encouraging. Shared antigens among the different mycobacterial species are the likely explanation for cross-reactive MAC immunity induced by BCG and DAR-901. Proteomic analysis of purified protein derivatives of *M. bovis* and *M. avium* showed that they share 16 proteins including secreted antigen Ag85C, Ag85B, and IdeR (iron-dependent repressor) [45]. Studies on peptides generated from selected protein clusters showed that the Mce family of proteins and a cluster of hypothetical proteins induce cross-reactive proliferative T cell responses [46]. Furthermore, mycobacterial lipids, although they may differ structurally across species [47], activate similar first-line pattern recognition receptors of the innate immune response [48,49].

Our results showed that mucosal BCG vaccination that targets the lungs is more protective against aerosolized MAC compared to systemic BCG or DAR-901 vaccination. This could be likely due to activation and recruitment of MAC-reactive T cells in lung mucosal tissues as shown in our histology results. Systemic administration of whole-cell vaccines may not provide the same level of antigens in the lung as mucosal vaccination to stimulate T cells relevant for controlling MAC growth [35]. In our study, we studied intradermal DAR-901 since that was the route used in animal and human studies for protection against TB [30,31,36,50].

BCG is a live-attenuated mycobacteria and mucosal delivery of BCG may not be safe in patients with underlying lung diseases who are at risk of developing pulmonary MAC [51,52]. One approach is the use of BCG with a “kill switch” (i.e., tetR BCG) as has been reported previously for TB [53]. We tested this approach using tetR BCG in our study. Two weeks of treatment of tetR BCG-vaccinated mice with doxycycline led to more than one log reduction in the number of lung BCG. Extended doxycycline treatment may reduce the lung BCG numbers more if BCG persists longer than 4 weeks after vaccination. Interestingly, doxycycline treatment did not interfere with MAC cross-reactive immunity induced by TetR BCG administered IN. Therefore, tetR BCG could be a potential safe approach for vaccination against pulmonary MAC when safety is a concern. Other approaches that could be tested in the future include tetR BCG followed by booster doses of DAR-901 or mucosal vaccination of DAR-901.

Because mucosal BCG vaccination induced the best protection against MAC, we studied lung inflammation and T cell activation or recruitment in mice vaccinated with IN BCG. These IN BCG-vaccinated mice had more inflammation compared to unvaccinated mice after the challenge with aerosolized MAC. This is likely due to the recruitment of effector T cells as shown by our flow cytometry results of lung T cells. After antigen encounters, T cells rapidly upregulate CD44 [54]. Therefore, CD44 is one of the most commonly used activation markers. CD44 expressing CD4 and CD8 T cells are activated effector T cells [55]). CD44 is important for a Th1 immune response [56], possibly by regulating survival and memory development [57]. In our study, we measured lung T cell responses only at two-time points (i.e., two and four weeks after MAC infection). Other groups, using C3HeB/FeJ mice, showed that the number of activated T cells peaked 40–50 days after MAC infection [58]. We also used CXCR3 staining to characterize lung T cells. CXCR3 is a chemokine receptor which plays an important role in T cell trafficking [59]. CXCR3 is rapidly induced on naïve cells following activation and remains highly expressed on Th1-type CD4^+^ T cells and effector CD8^+^ T cells. In our study, BCG-vaccinated mice had significantly increased frequencies of CD4^+^CXCR3^+^ T cells at 2 weeks but not at 4 weeks post-MAC infection. Our results also show that CXCR3 expression in CD8^+^ T cells at 2 or 4 weeks post-MAC infection did not change with BCG vaccination. The results may suggest that CD8^+^ are primarily activated in the lungs whereas CD4 T cells could be recruited to or activated in the lungs. Lung inflammation or recruitment of T cells is associated with decreased lung MAC burden as BCG-vaccinated mice had significantly lower numbers of MAC. It is important to study the progression of inflammation and MAC infection at multiple time points post-infection in vaccinated and unvaccinated mice. Our results comparing inflammation at 2 and 4 weeks after MAC infection indicate that inflammation in unvaccinated mice progresses, and this corresponds with an increasing number of MAC in the lungs. In future studies, we will characterize T cell responses following DAR-901 vaccination alone or in combination with BCG.

Macrolides, a key class of anti-MAC drugs including clarithromycin, are known to have anti-inflammatory activities [60,61]. The exact mechanisms determining how macrolides exert anti-inflammatory effects are not completely clear but it has been shown that macrolide treatment may lead to decreased levels of TNF-α and IL-12, cytokines which are important for the development of Th1 immunity [61]. It is also not clear how the macrolide anti-inflammatory properties are related to anti-MAC activity, or if interventions such as vaccinations which may lead to enhanced Th1 immunity affect the anti-MAC activity of macrolides. Our results clearly show that BCG vaccination does not interfere with the anti-MAC activity of clarithromycin administered for two weeks. In our study, we used the optimal dose of clarithromycin which was known to inhibit the growth of MAC by other groups [32,62,63]. Experiments with subinhibitory concentrations of MAC may further confirm the interactions of vaccine-induced immunity and anti-MAC or anti-inflammatory activities of macrolides.

## 5. Conclusions

Both DAR-901 and BCG vaccinations induce MAC cross-reactive immunity and protect against aerosolized MAC challenges. Mucosal BCG vaccination provides the best protection and is associated with increased inflammation following aerosol MAC infection and the presence of activated CD4^+^ and CD8^+^ T cells. There is concern that BCG, particularly mucosal BCG, may have the potential to cause harm in patients with structural lung diseases who are at risk of developing pulmonary MAC. TetR BCG could enhance the safety of mucosal BCG vaccination, but the duration of doxycycline treatment may need to be optimized. Two weeks of doxycycline treatment in mice decreased the numbers of tetR BCG by more than one log without affecting MAC protective immunity. Future studies should also include the effects of mucosal DAR-901 vaccination, booster effects of DAR-901 following vaccination with tetR BCG, and interactions of vaccine-induced MAC immunity with subinhibitory concentrations of macrolides.

## Data Availability

Research data is available from the corresponding author upon request.

## Supplementary Materials

The following supporting information can be downloaded at: https://www.mdpi.com/article/10.3390/vaccines13060619/s1, Table S1: Mouse types, intervention and MAC infection in different animal experiments. Figure S1: Outline of experiments on MAC immunity induced by DAR-901 compared to BCG. Figure S2: Outline of experiments that use whole cell vaccination for protection against MAC. Figure S3: Outline of experiments that use TetR BCG. Figure S4. *M. avium* cross reactive immunity induced by vaccination with BCG and DAR-901. Figure S5. Effects of BCG vaccinations on the growth of *M. avium* in lungs. Figure S6. Effector CD4 and CD8 T cells in the lung after aerosol MAC infection of vaccinated and unvaccinated mice. Figure S7. Effects of BCG vaccination on the anti-MAC effects of clarithromycin.
