# Supplementary material for: Immunity Against Mycobacterium avium Induced by DAR-901 and BCG
Source: Vaccines (Basel). 2025 Jun 7;13(6):619. doi: 10.3390/vaccines13060619 (PMC12197784; doi:10.3390/vaccines13060619)
Supplement: Supplementary file 1 [file vaccines-13-00619-s001.zip › vaccines-3667730-supplementary.pdf]

Supplementary Materials

Table S1. Mouse types, intervention and MAC infection in different animal experiments.

	Animal type and number of groups	Number of animals per group	Test item	Dose per vaccination/ intervention point	MAC challenge (aerosol)
Figure 1A	BALB/C, 8 groups	4	BCG ID<br>DAR-901	1 (1 x10^7 cfu)<br>2 and 3 doses of 0.3 mg, 1mg and 2 mg	-
Figure 1B	BALB/c, 3 groups	10	BCG ID<br>Dar-901 ID	1 (1 x10^7 cfu)<br>0.3 mg, 2 doses	-
Figure 2A	BALB/c, 5 groups	8	BCG ID<br>BCG SC<br>BCG IN<br>DAR-901	1 (1 x10^7 cfu)<br>1 (1 x10^7 cfu)<br>1 (1 x10^7 cfu)<br>0.3 mg, 2 doses	2 x10^7 cfu (all groups)
Figures 2B and 2C	BALB/c, 2 groups	5	BCG IN	1 (1 x10^7 cfu)	2 x10^7 cfu
Figure 3	BALB/c, 2 groups	5	BCG IN	1 (1 x10^7 cfu)	2 x10^7 cfu
Figure 4C	Scnn1b-Tg, 2 groups	5	TetR BCG IN<br>±Doxycycline	1 (1 x10^7 cfu)<br>1 mg/ml oral for 2 weeks	2 x10^7 cfu
Figure 5	Scnn1b-Tg, 3 groups	5	TetR BCG IN<br>±Doxycycline	1 (1 x10^7 cfu)<br>1 mg/ml oral for 2 weeks	2 x10^7 cfu
Supplementary Figure 7	BALB/c, 4 groups	BCG (n=5)<br>BCG+ drug (n=4)<br>Drug only (n=7)<br>No intervention (n=5)	BCG ID<br>Clarithromycin	1 (1 x10^7 cfu)<br>100 mg/kg gavage	2 x10^7 cfu

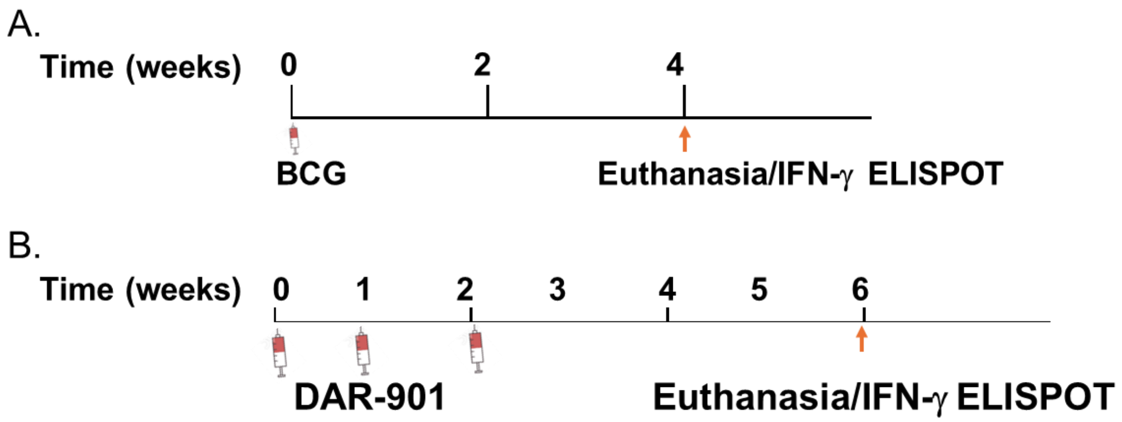

Supplementary Figure S1. Outline of experiments on MAC immunity induced by DAR-901 compared to BCG. One group of mice received BCG (ID). Different groups of mice were included for DAR-901 vaccinations (i.e., 2 and 3 doses of 0.3 mg, 1 mg, and 2 mg intradermally a week apart). Four weeks after BCG vaccination or four weeks after the last dose of DAR-901, mice were euthanized and splenocytes were used for IFN-γ ELISPOT assays with BCG and live MAC as antigens.

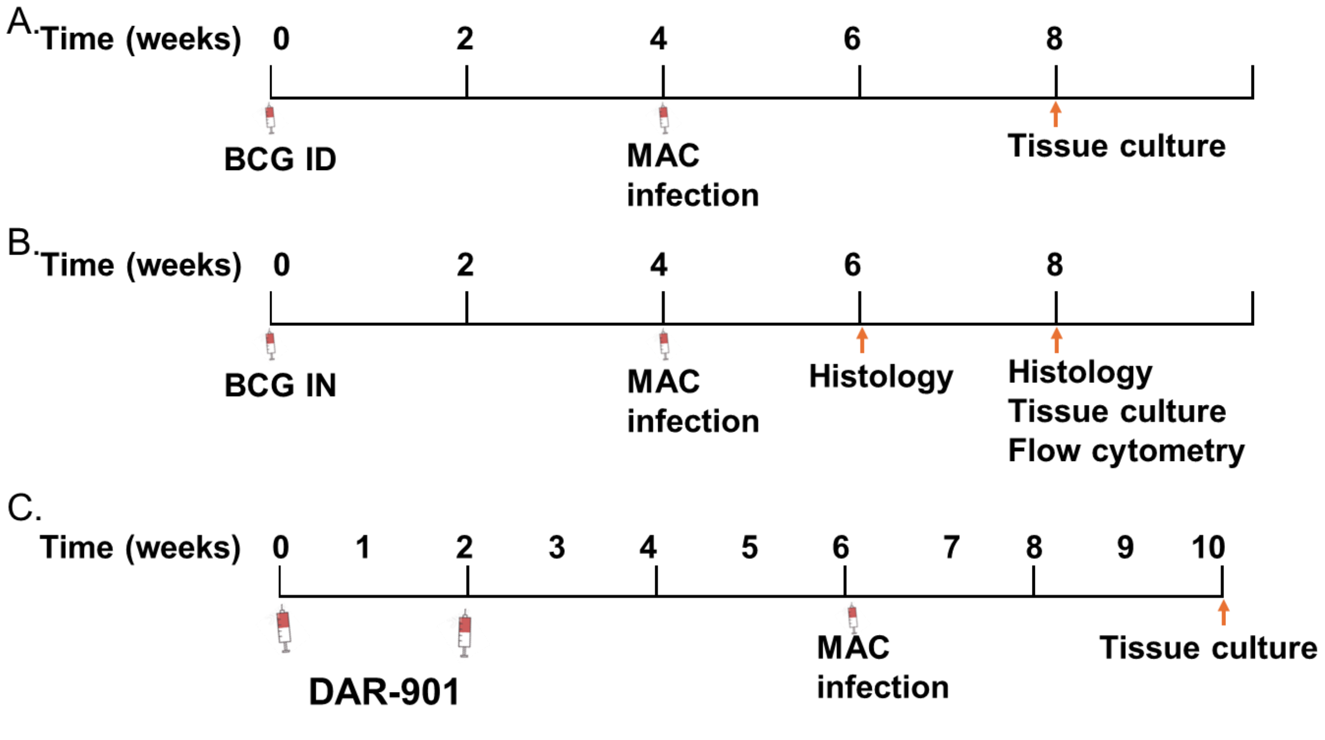

Supplementary Figure S2. Outline of experiments that use whole cell vaccination for protection against MAC. Three different vaccination routes or types were used. The first group received BCG ID (A), the second group received BCG IN (B) and the third vaccination group received 2 doses of DAR-901 2 weeks apart (c). Four weeks after BCG vaccination or the last dose of DAR-901, mice were infected with aerosolized MAC. For all groups, lung homogenates were cultured 4 weeks after MAC infection. For mice vaccinated with BCG ING, lung histology was performed at 2 and 4 weeks after MAC infection and lung cell flow cytometry was performed at 4 weeks after MAC infection.

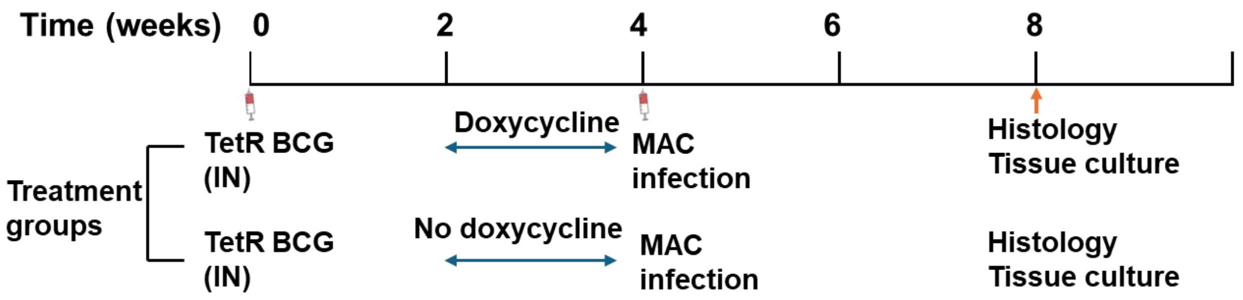

Supplementary Figure S3. Outline of experiments that use TetR BCG. Three groups of mice were used, two groups were vaccinated, and one group was kept unvaccinated. One of the vaccinated groups received doxycycline for 2 weeks starting two weeks after vaccination. All mice were infected with aerosolized MAC at 4 weeks post vaccination. Four weeks after MAC infection, lung homogenates were used for culture and lung was used for histology.

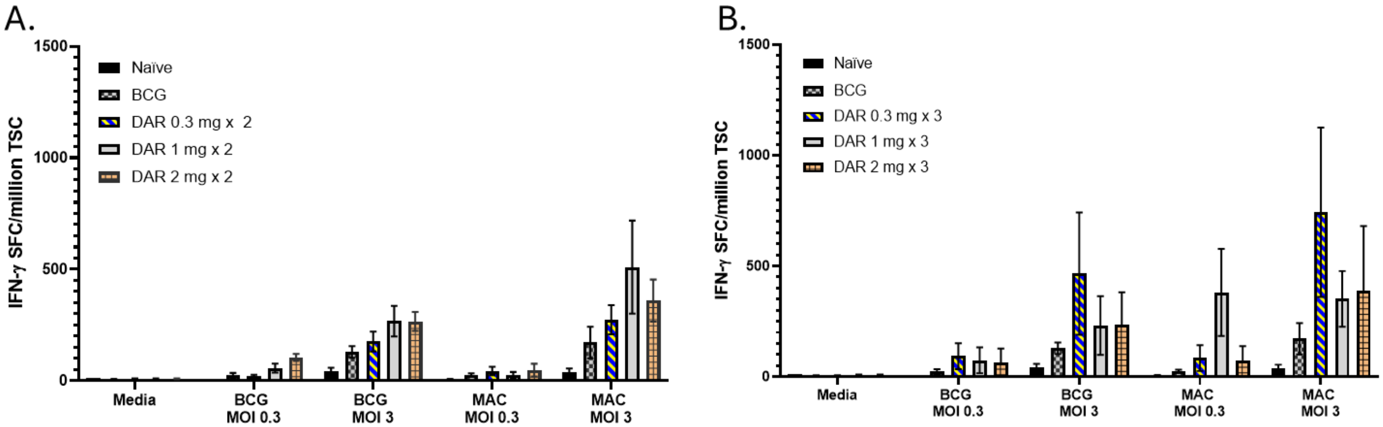

Supplementary Figure S4. *M. avium* cross reactive immunity induced by vaccination with BCG and DAR-901. Six-to 8-week-old female BALB/c mice were vaccinated with BCG (4 x 10^5 intradermal) or different concentrations of DAR-901 (2 or 3 intradermal doses 2 weeks apart). Four weeks after last vaccination, mice were euthanized and splenocytes were used for IFN-γ ELISPOT assay. In the ELISPOT assay, splenocytes (5 x 10^5 cells/well) were stimulated overnight with live BCG at multiplicity of infection (MOI) of 0.3 and 3, *M. avium* at MOI of 0.3 and 3, or media alone as a negative control. IFN-γ producing spots in each well were enumerated using a C.T.L. ImmunoSpot analyzer and software. The results are presented as spot forming cells (SFC, mean ± SE) per million total splenic cells (TSC). (A) shows results from mice vaccinated with BCG or 2 doses of DAR-901 a week apart at different concentrations. (B) shows results from mice vaccinated with BCG or 3 doses of DAR-901 a week apart at different concentrations.

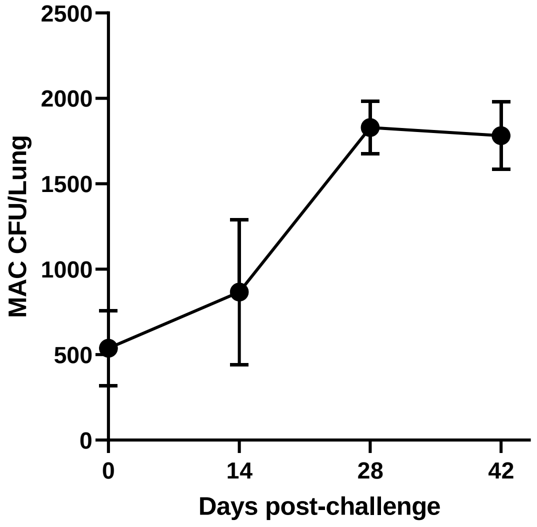

Supplementary Figure S5. Effects of BCG vaccinations on the growth of *M. avium* in lungs. A). Mice were infected with aerosolized *M. avium* at concentration of 2 x10^7 CFU/ml and euthanized on day 0, 14, 28 or 42. Homogenized lungs were cultured on 7H10 media. M. avium reaches peak growth 4 weeks after challenge.

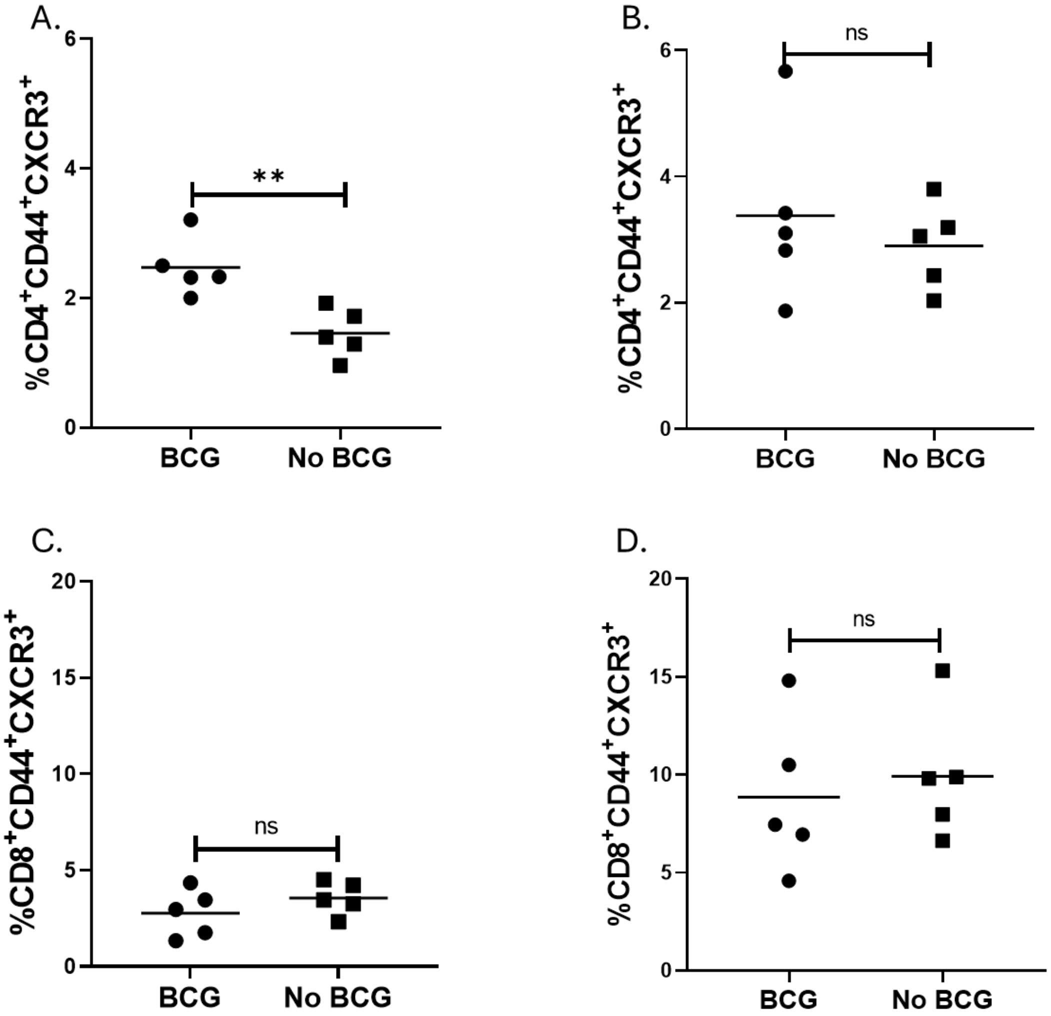

Supplementary Figure S6. Effector CD4 and CD8 T cells in the lung after aerosol MAC infection of vaccinated and unvaccinated mice. BALB/c mice vaccinated with IN BCG and unvaccinated mice were infected with aerosolized *M. avium* (ATCC700898, 2x 10^7 cfu/ml) 4 weeks after vaccination. Two- and four weeks post-infection (i.e., days 42 and 56 post-vaccination), lung tissues were homogenized. Cells were stained with live/dead, CD3, CD4, CD8, CD44 and CXCR3 antibodies. More than 10,000 events were acquired on flow cytometry. A) and B) show percent $CD4^+/CD44^+/CXCR3^+$ T cells in vaccinated and unvaccinated mice on days 42 and 56, respectively. C) and D) show percent $CD8^+/CD44^+/CXCR3^+$ T cells in vaccinated and unvaccinated mice on days 42 and 56, respectively. The results show that vaccinated mice had significantly higher percent $CD4^+/CD44^+/CXCR^+$ T cells two weeks after MAC infection (p= 0.0079, Mann Whitney U test), but not at 4 weeks post-infection (p= 0.84). Vaccinated and unvaccinated mice in the percent of CXCR expressing $CD8^+/CD44^+$ T cells with p= 0.34 and 0.69 at two-and four- weeks post-infection, respectively.

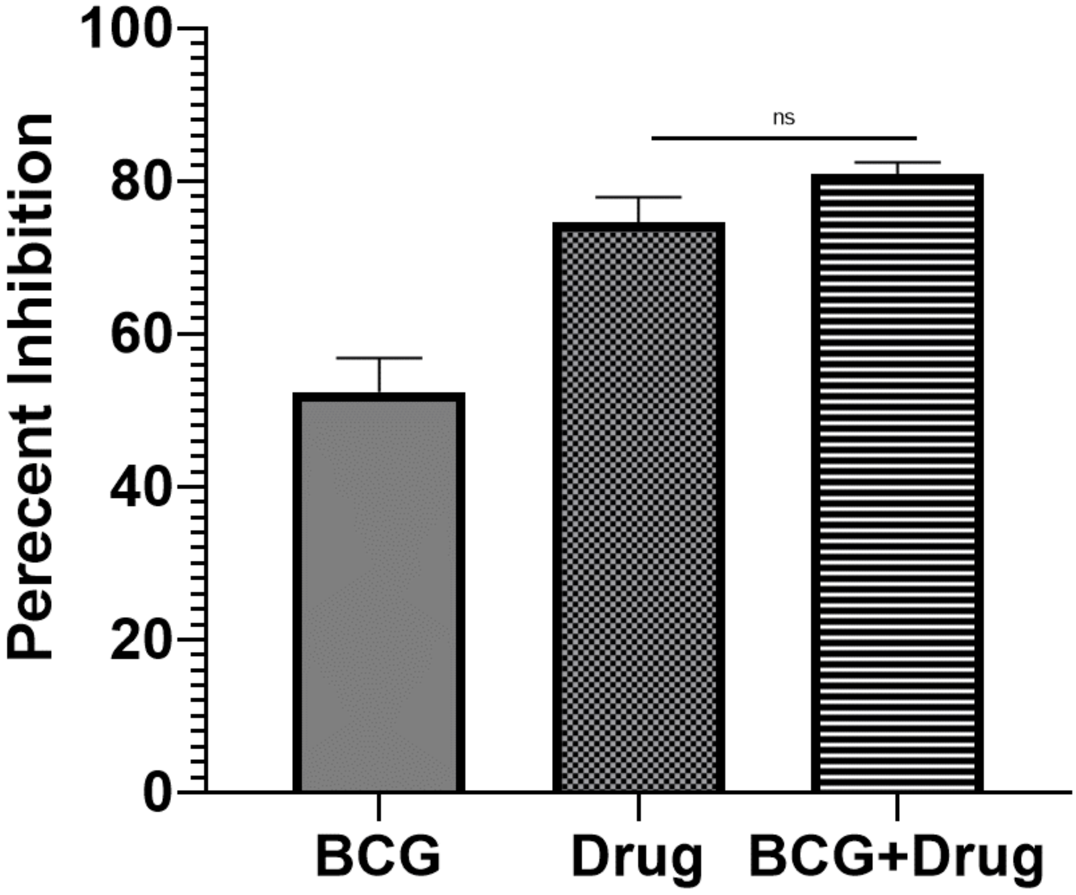

Supplementary Figure S7. Effects of BCG vaccination on the anti-MAC effects of clarithromycin. Nine BALB/c mice were vaccinated with BCG SC (1 x 10^7 bacteria in 100 μL to base of tail) and twelve mice were kept unvaccinated. Four weeks after vaccination, all mice were infected with aerosolized MAC at a concentration of 2 x10^7 CFU/ml. Two weeks after infection, four BCG-vaccinated and seven unvaccinated mice received clarithromycin at a concentration of 2 mg per 20 g via gavage 5 days a week. All mice were euthanized six weeks after infection, lungs homogenized and CFU quantified by culturing on 7H10 media. SC BCG did not interfere with anti-MAC activities of clarithromycin.
